# Open Access: hearing the music but not knowing the lyrics. The evolution of knowledge and perceptions among researchers at a Spanish university (2021–2024)

**DOI:** 10.12688/openreseurope.24242.1

**Published:** 2026-07-06

**Authors:** Ramón A. Feenstra, Emma Gómez Nicolau

**Affiliations:** 1 Philosophy and Sociology, Universitat Jaume I Facultat de Ciencies Humanes i Socials, Castellón, Valencian Community, 12071, Spain

**Keywords:** Open Access, Open Science Literacy, Repositories, Science Policy, University, Spain

## Abstract

After years of advocacy in favour of Open Access (OA), it is a timely moment to assess whether knowledge of its meaning and practical implications has become truly established within a specific research community. This study analyses the evolution of attitudes and knowledge concerning OA among the staff at the Universitat Jaume I (UJI) between 2021 and 2024—a period marked by regulatory reforms in Spain and a significant rollout of institutional policies. To this end, we examine the results of two institutional surveys conducted in 2021 (N = 505) and 2024 (N = 330), categorising participants by academic career stage (R1–R4), field of knowledge, and gender. The study evaluates critical dimensions such as knowledge of OA pathways, the use of the institutional repository, and research data management. Our findings suggest that, although familiarity with the concept is increasing, the research community remains far from mastering the practical implications of Open Access. These data provide critical evidence regarding the effectiveness of Open Science policies in transforming academic culture.

## Introduction

Since the beginning of the 21st century, the promotion of Open Access (OA) has been driven by a series of international declarations. The Budapest Open Access Initiative (2002), the Bethesda Statement on Open Access Publishing ( Brown et al., 2003), and the Berlin Declaration on Open Access to Knowledge ( Max Planck Society, 2003) defined the key principles aimed at promoting unrestricted access to research outputs. These declarations were later reinforced by initiatives such as Plan S ( cOAlition S, 2018) and the Open Access policies of major European and national funding agencies.

The objective of these movements was to promote the dissemination of results; to achieve this, it is essential to transform the dynamics of the research community, transitioning from a publication culture centred on the prestige economy (and individual curricula) towards a model of science oriented towards social return ( Moher et al., 2020). After two decades of these movements, a critical question arises: to what extent have these principles truly permeated the scientific community? Do researchers really understand what Open Access means and—above all—are they aware of its practical implications?

The aim of this study is to analyse the attitudes and self-reported knowledge of researchers at a specific Spanish university, the Universitat Jaume I (UJI), regarding Open Access. This context is of particular interest given that both national and university-level policies have been directed towards promoting Open Access to research (and knowledge of its development). Thus, this analysis allows for an evaluation of whether these efforts have taken root in the organisational culture and the mindset of a research community, or if, conversely, Open Access is perceived as somewhat detached from the research lifecycle.

## Between conceptual adhesion and the technical gap: Dimensions of open access in the literature

The body of literature includes a broad and growing body of empirical studies focused on the perceptions, attitudes, and practices of researchers regarding Open Access. These works explore various national contexts, including, among others, Bulgaria ( Boock et al., 2020), the United Kingdom ( Zhu, 2017), the United States ( Yang & Li, 2015), Canada ( McDonald et al., 2016), India ( Dhanavandan & Tamizhchelvan, 2013), Pakistan ( Sheikh, 2019), the United Arab Emirates ( Kaba & Said, 2015), Israel ( Aharony & Hadad, 2022), Nigeria ( Obuh & Bozimo, 2012), and Australia ( Narayan et al., 2018). To this literature, global-scale works can be added, such as those by Dallmeier-Tiessen et al. (2011) and Taylor & Francis (2019). In the Spanish context, a robust body of research has analysed the evolution of the scientific community towards the Open Access paradigm through various approaches ( Hernández-Borges et al., 2006; Serrano-Vicente et al., 2016; Ruiz-Pérez & Delgado-López-Cózar, 2017; Segado-Boj et al., 2018; González-Teruel et al., 2022; Abad García et al., 2022). Taken together, this literature allows for the comparison of realities across different levels of regulatory and institutional development.

These studies have focused on several dimensions, including aspects such as: (a) attitudes and perceived reputation of Open Access journals ( Serrano-Vicente et al., 2016; Zhu, 2017; Segado-Boj, Martín-Quevedo & Prieto-Gutiérrez, 2018; Sheikh, 2019; Taylor & Francis, 2019; Boock et al., 2020); (b) knowledge or familiarity, both general—regarding the concept itself ( Schroter & Tite, 2006; Obuh & Bozimo, 2012; Dhanavandan & Tamizhchelvan, 2013; Rodriguez, 2014; Yang & Li, 2015; Kaba & Said, 2015; Ruiz-Pérez & Delgado-López-Cózar, 2017; Boock et al., 2020)—and specific, concerning the different Open Access routes ( McDonald et al., 2016; Segado-Boj, Martín-Quevedo & Prieto-Gutiérrez, 2018; Narayan et al., 2018; Aharony & Hadad, 2022), as well as repositories ( Hernández-Borges et al., 2006; Obuh & Bozimo, 2012; Yang & Li, 2015; Narayan et al., 2018; Sheikh, 2019; Aharony & Hadad, 2022); (c) research community practices regarding Open Access ( Rowley et al., 2017; Narayan et al., 2018; Taylor & Francis, 2019); (d) the weight of Open Access as a selection criterion for journals in which to publish ( Rodriguez, 2014; McDonald et al., 2016; Narayan et al., 2018; Boock et al., 2020); and (e) positioning regarding the payment of APCs ( Schroter & Tite, 2006; McDonald et al., 2016; Taylor & Francis, 2019). Furthermore, this literature on Open Science has examined and documented existing differences between disciplinary fields ( Taylor & Francis, 2019), as well as discernible differences in knowledge, attitudes, or assessments of Open Access based on gender ( Kaba & Said, 2015) or academic age ( Serrano-Vicente et al., 2016; Zhu, 2017; Segado-Boj, Martín-Quevedo & Prieto-Gutiérrez, 2018).

Despite this thematic and contextual breadth and diversity in Open Access studies, the literature converges on a widely documented trend in both individual studies and comprehensive reviews ( Togia & Korobili, 2014): specific and detailed knowledge of the operational implications of Open Access continues to exhibit certain deficiencies. Literacy regarding routes, repositories, and support tools remains rather limited and lower than the general perception of what Open Access represents. To simplify this with a metaphor, it could be said that the research community appears to recognise the melody or the music, but not always the lyrics that accompany it. That is, there is a certain conceptual familiarity and favourable predisposition, yet a notable lack of knowledge when it comes to integrating practical implications into research routines.

## Spain and the universitat jaume i as a research context

The present research offers an ideal framework to further investigate this potential gap between familiarity and actual knowledge. It analyses the Spanish context—which has consistently promoted Open Access through public policies—and the Universitat Jaume I (UJI), which has implemented various actions to promote these policies and their understanding.

Regarding the national framework, Law 14/2011 on Science, Technology, and Innovation, reformed by Law 17/2022, recognises Open Access as a structural principle of research activity and establishes clear obligations for open dissemination. This is further supported by the Organic Law 2/2023 on the University System (LOSU), which reinforces the role of universities in scientific outreach, emphasising responsible evaluation, accountability, and openness. Finally, this process has been consolidated by the National Open Science Strategy (ENCA, 2023–2027) ^1^, the most ambitious state framework to date for driving Open Access, FAIR data, responsible evaluation, and citizen participation.

Furthermore, this regulatory rollout has found its most tangible application in research practice through the recent reform of the evaluation frameworks of the National Agency for Quality Assessment and Accreditation (ANECA). These are aligned with the principles of the CoARA coalition, which seeks to transform scientific assessment models. In this context, a fundamental milestone was the 2023 *sexenios* call—a national scheme that recognises research performance through peer-review assessment every six years— which for the first time integrated “Contribution to Open Science” as a specific criterion with a 10% weight in the scoring. This involves the assessment of aspects such as depositing in repositories, the use of the diamond route, or Creative Commons licences. This transformation was further solidified on 1 April 2024, with the commencement of the new state accreditation system (derived from RD 678/2023), which incorporates a more qualitative assessment. Through these measures, the Spanish evaluation system attempts to align career incentives with the values of Open Science.

In this scenario, the Universitat Jaume I (UJI) has sought to align itself with these requirements through its institutional policies. In recent years, it has hosted two European projects, ETHNA System (2020–2023) and CATALISI (2023–2025), as catalysts for promoting Open Access. These initiatives have crystallised into concrete governance actions driven by the Vice-Rectorate for Research, such as the creation in 2021 of a Code of Good Practice in Research ^2^—with a specific section on Open Access—and its subsequent update in 2025 to integrate new commitments regarding Open Science. Other actions include the development of specific training courses on Open Access ^3^, the publication of an “Open Access thermometer” ^4^ to monitor the university’s open output, and, finally, the definition of an awareness programme within the ENCA-UJI 2024–2027 plan ^5^. The two surveys underpinning this study (2021 and 2024) were conducted within the framework of these projects, designed to diagnose the initial perceptions of the university community and to examine the evolution resulting from university policies. The value of this work lies in the possibility of comparing two points in time (2021 and 2024) that capture a critical phase of this ongoing institutional transformation. If, as the literature suggests ( Moore et al., 2017), cultural changes are complex processes that must overcome both external (incentives) and internal (literacy) barriers, the UJI case allows us to address a fundamental question: has knowledge of the “lyrics” of Open Access improved after these institutional efforts, or does the scientific community still only hear the “melody”?

## Methodology

This study analyses two surveys conducted at the Universitat Jaume I (UJI) in 2021 and 2024, aimed at Teaching and Research Staff (PDI) and Research Staff (PI). In both cases, the surveys were administered via the Qualtrics platform, available in Valencian and Spanish (and additionally in English in 2024), and included questions on Open Access and Open Science, alongside other relevant areas of research culture.

The first survey, conducted between 5 May and 13 June 2021, was part of an institutional project on research ethics linked to the European ETHNA System project. It comprised five thematic blocks, of which this article analyses the block relating to Open Access. The total population was 1,030 researchers. A total of 505 valid responses were received (49.03%), with a sampling error of ±3.18%. The second survey, conducted between 25 November and 22 December 2024, was framed within the institutional deployment plans for the National Open Science Strategy (ENCA) and responsible assessment (CoARA), linked to the European CATALISI project. The questionnaire consisted of 36 items grouped into four blocks, of which this article analyses the Open Science block. The surveyed population consisted of 976 members of the PDI/PI, yielding 330 valid responses (33.81%), with a sampling error of ±4.39%. The slightly lower population figure compared to 2021 is due to a reduced presence of research staff and certain scholarship programmes in the 2024 institutional census. For both survey iterations, informed consent was obtained electronically from all participants prior to their participation. Before accessing the online questionnaires, respondents were presented with an introductory sheet detailing the study’s purpose, data confidentiality protocols, and compliance with the General Data Protection Regulation (GDPR). Participants provided their explicit consent by actively agreeing to these terms to proceed to the survey questions. The anonymity of responses was strictly maintained throughout, and data were processed in an aggregated manner exclusively for academic research purposes and institutional policy development.

In both surveys, participants were classified according to the European R1–R4 categories ( Table 1) and five broad fields of knowledge (Arts and Humanities, Sciences, Health Sciences, Social and Legal Sciences, and Engineering and Architecture; Table 2). The two surveys together allow for a comparative examination of the evolution of knowledge, attitudes, and practices in Open Access over an interval marked by profound regulatory and institutional transformations.

**Table 1.** Comparison of research population and survey sample by professional category (2021 vs. 2024).

Professional Category	2021 Pop (N)	2021 Sample (n)	2021 Resp. Rate	2024 Pop (N)	2024 Sample (n)	2024 Resp. Rate
**R1** (Pre-doctoral)	243	52	21.4%	178	34	19.1%
**R2** (Post-doctoral)	169	89	52.7%	209	75	35.9%
**R3** (Established)	448	259	57.8%	392	142	36.2%
**R4** (Leading)	170	105	61.8%	197	79	40.1%
**Total**	**1,030**	**505**	**49.0%**	**976**	**330**	**33.8%**

**Table 2.** Comparison of research population and survey sample by field of knowledge (2021 vs. 2024).

Field of Knowledge	2021 Pop (N)	2021 Sample (n)	2021 Resp. Rate	2024 Pop (N)	2024 Sample (n)	2024 Resp. Rate
Arts and Humanities (AH)	127	58	45.7%	126	52	41.3%
Sciences (S)	194	95	49.0%	201	62	30.8%
Health Sciences (HS)	34	25	73.5%	39	18	46.2%
Social and Legal Sciences (SLS)	393	202	51.4%	391	125	32.0%
Engineering and Architecture (EA)	282	125	44.3%	219	73	33.3%
**Total**	**1,030**	**505**	**49.0%**	**976**	**330**	**33.8%**

Statistical tests indicated that the 2024 sample maintained the population structure across branches of knowledge. Internal validation also confirmed representativeness regarding gender balance—although this variable was subsequently omitted from the shared dataset to protect participant anonymity—however, this was not the case for professional categories.

Prior to their launch, both surveys underwent an initial testing phase. The pilot versions were reviewed and commented upon by a working group consisting of 13 members of the university community in 2021 (AH: 3, HS: 2, S: 2, SLS: 4, and 2 research technicians) and 12 members in 2024 (AH: 2, HS: 1, S: 3, SLS: 4, EA: 2). Gender parity was also sought (six men and seven women in 2021; five men and seven women in 2024).

Regarding the research instrument, both surveys shared a common structure that enables the analysis of researchers’ perceptions and practices concerning Open Access. Specifically, both instruments include items on the level of knowledge of Open Access routes, the use of the institutional repository, the capacity to develop Data Management Plans (DMPs), and the importance attributed to Open Access publishing or its consideration within journal selection criteria.

Furthermore, the 2024 survey represents a substantial methodological reinforcement of the Open Science block, evolving from the exploratory nature of 2021 towards a more in-depth and multidimensional diagnosis. This expansion responds to the need for an updated empirical basis to inform the revision of the Code of Good Practice in Research (2025) and the redesign of institutional training plans. Consequently, the 2024 instrument introduces a specific battery of questions on attitudes and values towards Open Access and increases the technical granularity of the enquiry. This involves an expansion of questions and response options regarding journal selection factors (reputation, APC costs, editorial speed, or acceptance rates), as well as the technical distinction between access routes and figures such as preprints and postprints.

Taken together, the shared structure allows for a direct comparison of a portion of the results between 2021 and 2024, while the thematic expansion of the 2024 questionnaire enriches the analysis by capturing emerging dimensions of the Open Science ecosystem. This provides a more nuanced reading of researchers’ practices, knowledge, and interpretations, albeit making comparison more complex. The following table describes the types of variables studied in both surveys and the comparability and analysis strategy employed ( Table 3).

**Table 3.** Comparison of survey variables and instrument comparability.

Survey 2021	Survey 2024	Comparability
**PIM (**Personal Importance): Likert-type item (1–5) - How much importance do you attach to your own research being disseminated via Open Access?	**FOA** (Free Online Access): Likert-type item (1–5) - All research findings should be available to read online free of charge. **NMD** (Non-Monetised Dissemination): Likert-type item (1–5) - Research dissemination is a common good and should not be monetised in any way. **DAP** (Data over Articles Preference): Likert-type item (1–5) - Open access to data is more important to me than open access to research articles. **INN** (Innovation Driver): Likert-type item (1–5) - Open access drives innovation in research. **NFA** (No Fundamental Advantages): Likert-type item (1–5) - Open access publishing offers no fundamental advantages.	While the 2021 survey established a baseline for scholarly communication trends, the 2024 iteration introduces a specific battery of five attitudinal variables (FOA, NMD, DAP, INN, NFA) alongside a personal importance metric (PIM). It should be noted that these new variables are not intended for longitudinal comparison with the 2021 dataset. Instead, they have been incorporated to broaden the analytical scope and capture a more nuanced profile of researchers’ values.
**Main Question:** *When selecting a journal or publisher for your work, please indicate which factors you take into account (select all that apply):* **OA_PUB** (Open Access availability): Binary item (Yes/No) - The work is published via open access. **CIT_IMP** (Citation impact): Binary item (Yes/No) - Impact based on the number of citations. **PRE_TRAD** (Prestige and tradition): Binary item (Yes/No) - The prestige and tradition of the publication. **PUB_SPD** (Publication speed): Binary item (Yes/No) - The speed of the publication process. **REV_QUAL** (Peer-review quality): Binary item (Yes/No) - The quality of the selection and peer-review process. **SUB_SPEC** (Subject specialisation): Binary item (Yes/No) - The thematic orientation or specialisation of the publisher. **VIS_DB** (Database visibility): Binary item (Yes/No) - Dissemination and visibility of the journal in databases. **NO_APC** (Absence of fees): Binary item (Yes/No) - No publication fees or charges are required.	**Main Question:** *How important are the following factors when deciding where to publish your work?* 1 = Not at all important, 5 = Extremely important **REP_BRD** (Editorial Reputation): Likert-type item (1–5) – The journal has a good reputation in my field (e.g. a good editor and editorial board). **HGH_IF** (High Impact Factor): Likert-type item (1–5) – The journal has a high impact factor in my field. **WID_AUD** (Wide Audience): Likert-type item (1–5) – The journal is read by a wider audience. **DIA_OA** (Diamond Open Access): Likert-type item (1–5) – The journal is fully open access and all articles published are available at no cost to authors and readers. **COL_PUB** (Colleague Publications): Likert-type item (1–5) – My colleagues have published contributions in the journal. **SHR_TAT** (Short Turnaround Time): Likert-type item (1–5) – The journal is known for its short turnaround times. **HYB_APC** (Hybrid APC Option): Likert-type item (1–5) – The journal has the option of making the content available in open access, upon payment of article processing charges (APC). **ACC_RATE** (Acceptance Rate): Likert-type item (1–5) – The article acceptance rate.	The 2024 instrument incorporates updated items to align with current trends in the international scholarly communication literature. A significant methodological refinement was the transition from dichotomous (Yes/No) scales to importance-based Likert scales. This shift was implemented to enhance the granularity of the data, allowing for more robust statistical analyses, such as the calculation of means and variances. Consequently, while direct longitudinal comparison is limited, comparability is achieved through relative hierarchical ordering: the 2021 factors are ranked by frequency, whereas the 2024 factors are ranked by mean scores. This approach requires an interpretive analysis of the relative importance assigned to each factor across both periods, prioritising thematic consistency over direct numerical matching.
**ROUTE** (Routes Knowledge): Likert-type item (1–5) – How would you rate your level of knowledge of the different routes (green, gold, bronze, hybrid or diamond/platinum) to Open Access publishing? 1 = very low; 5 = excellent	**ROUTE** (Routes Knowledge): Likert-type item (1–5) – How would you rate your level of knowledge of the different routes (green, gold, bronze, hybrid or diamond/platinum) to open access publishing? 1 = very low; 5 = excellent	The question remains identical, ensuring statistical comparability.
**REP** (Repository Knowledge): Likert-type item (1–5) – How would you rate your level of awareness about the UJI repository and the possibilities it offers for disseminating your research? 1 = very low; 5 = excellent	**REP** (Repository Knowledge): Likert-type item (1–5) – How would you rate your level of awareness about the UJI repository and the possibilities it offers for disseminating your research? 1 = very low; 5 = excellent	The question remains identical, ensuring statistical comparability.
**DMP** (DMP Knowledge): Likert-type item (1–5) – How would you rate your level of awareness when it comes to developing or designing a data management plan for a research project? 1 = very low; 5 = excellent	**DMP** (DMP Knowledge): Likert-type item (1–5) – How would you rate your level of awareness when it comes to developing or designing a data management plan for a research project? 1 = very low; 5 = excellent	The question remains identical, ensuring statistical comparability.

The 2024 survey incorporated new variables that enable a more in-depth exploration of perceptions and practices regarding Open Science ( Table 4). Specifically, the following were adopted:

**Table 4.** Extra survey variables in the 2024 survey.

**Main Question:** *Awareness of Open Access (specific by routes). How would you rate your level of knowledge for each of the routes?* GOLD (Gold): Likert-type item (1–5) - Gold DIA (Diamond): Likert-type item (1–5) - Diamond/platinum GRE (Green): Likert-type item (1–5) - Green BRO (Bronze): Likert-type item (1–5) - Bronze HY (Hybrid): Likert-type item (1–5) - Hybrid 1 = very low; 5 = excellent
**PRE/POST** (Preprint/Postprint Knowledge): Likert-type item (1–5) - How would you rate your level of awareness of concepts such as “preprint/postprint”? 1 = very low; 5 = excellent
**COMM** (Open Science Comments): Open-ended text - Please feel free to use the space below to share your personal opinion and assessment of this block on Open Science, if you wish to do so.

Statistical software SPSS (v.29) was utilised for data processing. The analysis was structured across three levels to ensure the robustness of the findings:

1. Descriptive Analysis: Frequencies and percentages were calculated for the nominal variables of the 2021 instrument. For the 2024 Likert scale, measures of central tendency were employed, primarily the arithmetic mean ( *M*).
2. Parametric Inferential Analysis: To compare two independent groups (gender variable), the Student’s *t*-test was applied. For comparisons involving three or more groups (professional category and field of knowledge), one-way analysis of variance (ANOVA) was performed.
3. Effect Size and Significance: The level of statistical significance was set at *p* < .05. Effect sizes are reported to quantify the practical or clinical relevance of the differences: Cohen’s *d* was used for *t*-tests and η ^2^ (Eta-squared) for the analysis of variance.

Finally, the qualitative analysis of the open-ended question was conducted using thematic analysis. Quotes are identified by the participant’s registered gender (F for Female, M for Male), field of research, and research career stage (R1–R4).

## Results

### Evolution of perceptions regarding open science (2021–2024)

The first aspect to analyse in the evolution between 2021 and 2024 is the relevance associated with the open dissemination of research; that is, the desirability of consolidating Open Access (OA). In 2021, this issue obtained a mean score of 4.00, demonstrating very notable support for the ideal of making research results open.

The 2024 instrument has allowed for a more in-depth exploration of attitudes towards Open Access and the relevance accorded to it, thanks to the inclusion of a battery of variables (which go beyond merely asking about the importance of OA). The results once again confirm that support is widespread. It is worth noting that, regarding the item assessing OA as a common good, there is almost complete consensus that access to research should be free of charge (FOA: *M* = 4.58) and non-monetisable (NMD: *M* = 4.49). Conversely, it has also been observed that free access to data (DAP: *M* = 3.07) generates significantly less adhesion than access to articles.

However, the relevance assigned to OA is conditioned by demographic and professional factors. Along these lines, even in 2021, the positive evaluation of OA was not uniform. The Student’s t-test for independent samples highlighted the existing gender gap. The importance attributed to open dissemination was significantly higher among women ( *t*(517) = −4.050; *p* < .001), with an effect size of *d* = −0.357). Regarding professional category, a downward linear trend was observed: early-stage researchers (R1: *M* = 4.23) valued Open Access more highly than established profiles (R4: *M* = 3.85), with statistically significant differences ( *F* (3, 501) = 3.63; *p* = .013; η ^2^ = .021). Finally, within the 2021 survey ( *p* = .439), no significant differences were found regarding fields of knowledge ( Table 5).

**Table 5.** Personal importance awarded to OA. 2021. Means by gender, research area and career stage.

	Mean	Men	Women	AH	S	HS	SLC	EA	R1	R2	R3	R4
**PIM** (Personal Importance)	4.00	3.86	4.18	4.03	3.87	4.08	4.07	3.97	4.23	4.17	3.95	3.86

In 2024, these patterns exhibited substantial variations across several items. The results showed that women held a significantly more common good-oriented vision than men. This was particularly evident in the variable NMD (“Research is a common good and should not be monetised”), where the difference was highly significant ( *t* (325) = −3.518; *p* < .001). The effect size for this item was among the highest in the study ( *d* = −0.389), which is considered a small-to-medium but very robust effect in terms of trend. Furthermore, women had a stronger perception of Open Access as a driver of innovation (INN: *p* < .001; *d* = −0.371) and argued that results should be free of charge for the reader (FOA: *p* = .022; *d* = −0.255). The analysis of the variable NFA (“Open Access publishing has no fundamental advantages”) was especially revealing, as it showed the only reversal of the general trend ( *t* (326) = 1.974; *p* = .049); in this case, it was men who showed significantly higher agreement with this sceptical view.

Regarding professional category, changes were observed in 2024 compared to 2021, with the intermediate profiles (R2 and R3) defending non-monetisation most intensely ( *M* > 4.60). Conversely, senior profiles (R4) showed the lowest degree of agreement regarding free access for readers ( *M* = 4.35), and the previously observed downward linear trend was no longer present. At the initial stage (R1), there was less support for free access. Statistically significant differences were identified according to researcher category ( *F* (3, 325) = 3.357; *p* = .019), with an effect size of η ^2^ = .030 for free access (FOA). Similarly, Research as a Non-monetisable Common Good (NMD) yielded a highly significant result ( *F* (3, 323) = 3.975; *p* = .008), with an associated effect size of η ^2^ = .036.

When examining fields of knowledge, the 2024 ANOVA revealed that branch of research explained nearly 6% of the variability in the conception of research as a common good (NMD: η ^2^ = .059). The belief that research should be free to access was highest in Health Sciences (FOA: *M* = 5.00), while the view that it should be non-monetisable was most prominent in Arts and Humanities (NMD: *M* = 4.86). These positions differed significantly from those held in the Sciences (FOA: *M* = 4.39; NMD: *M* = 4.24) and Engineering (FOA: *M* = 4.49; NMD: *M* = 4.28) ( Table 6).

**Table 6.** Attitudes and values regarding research communication. 2024. Means by gender, research area and career stage.

	Mean	Men	Women	AH	S	HS	SLC	EA	R1	R2	R3	R4
**FOA** (Free Online Access)	4.59	4.49	4.69	4.83	4.39	5.00	4.58	4.49	4.74	4.68	4.63	4.35
**NMD** (Non-Monetised Dissemination)	4.50	4.33	4.68	4.87	4.24	4.76	4.56	4.28	4.24	4.64	4.62	4.27
**DAP** (Data over Articles Preference)	3.08	3.07	3.09	3.00	2.89	3.22	3.15	3.14	3.18	3.20	3.07	2.92
**INN** (Innovation Driver)	4.41	4.25	4.58	4.40	4.16	4.67	4.44	4.51	4.65	4.43	4.47	4.19
**NFA** (No Fundamental Advantages)	1.70	1.82	1.58	1.58	2.02	1.33	1.58	1.81	1.85	1.68	1.59	1.85

### Evolution of publication criteria (2021–2024)

Another relevant dimension in investigating the weight or significance of Open Access consists in enquiring into the criteria researchers consider a priority when selecting a journal or publisher for their findings. It is worth noting that between 2021 and 2024, an evolution occurred in the nature of the data collected: while prevalence (frequency of selection) was measured in 2021, intensity (degree of importance on a 1–5 Likert scale) was evaluated in 2024. Despite this methodological transition, the results indicated a clear hierarchy in the academic community’s priorities ( Table 7).

**Table 7.** Comparative analysis of publication criteria: Prevalence (2021) vs. Intensity (2024).

2021 (Prevalence)	2024 (Intensity)
**CIT_IMP** (Citation impact): Impact based on the number of citations. (75.7%)	**HGH_IF** (High Impact Factor): The journal has a high impact factor in my field. ( *M* = 4.6091)
**PRE_TRAD** (Prestige and tradition): Prestige and tradition of the publication. (58.3%)	**REP_BRD** (Editorial Reputation): The journal has a good reputation in my field (e.g. a good editor and editorial board). ( *M* = 4.3758)
**SUB_SPEC** (Subject specialisation): The thematic orientation or specialisation of the publisher. (46.9%)	**DIA_OA** (Diamond Open Access): The journal is fully open access and all articles published are available to authors and readers at no cost. ( *M* = 3.7781)
**NO_APC** (Absence of fees): No publication fees or charges are required. (41.0%)	**WID_AUD** (Wide Audience): Likert-type item (1–5) – The journal is read by a wider audience. ( *M* = 3.6201)
**PUB_SPD** (Publication speed): The speed of the publication process. (36.7%)	**SHR_TAT** (Short Turnaround Time): The journal is known for its short turnaround times. ( *M* = 3.3689)
**OA_PUB** (Open Access availability): The work is published via open access. (34.5%)	**ACC_RATE** (Acceptance Rate): The article acceptance rate. ( *M* = 3.0091)
**VIS_DB** (Database visibility): Dissemination and visibility of the journal in databases. (30.4%)	**HYB_APC** (Hybrid APC Option): The journal has the option of making the content available in open access, upon payment of article processing charges (APC). ( *M* = 2.7829)
**REV_QUAL** (Peer-review quality): The quality of the selection and peer-review process. (29.5%)	**COL_PUB** (Colleague Publications): My colleagues have published contributions in the journal. ( *M* = 2.7829)

The data in the table reveal stability in the pillars of the incentive system, as observed in the prevalence of “High Impact Factor” and “Reputation/Prestige”, which remain the dominant factors in both periods. However, it is crucial to note the evolution of the academic community’s positioning regarding OA: while in 2021 it was a secondary criterion (34.5%), in 2024 it achieved a notable score ( *M* = 3.78).

The analysis of the 2024 survey allows for a more in-depth exploration of the sociodemographic and professional variables that modulate these results, revealing several noteworthy data points ( Table 8).

**Table 8.** Publication criteria. 2024. Means by gender, research area and career stage.

	Mean	Men	Women	AH	S	HS	SLC	EA	R1	R2	R3	R4
**REP_BRD** (Editorial Reputation)	4.38	4.25	4.51	4.54	4.34	4.33	4.42	4.22	4.47	4.27	4.36	4.47
**HGH_IF** (High Impact Factor)	4.61	4.55	4.67	4.69	4.56	4.83	4.55	4.63	4.68	4.71	4.63	4.46
**WID_AUD** (Wide Audience)	3.62	3.45	3.80	3.69	3.45	3.67	3.67	3.62	3.65	3.61	3.65	3.56
**DIA_OA** (Diamond Open Access)	3.78	3.58	3.99	4.21	3.33	4.11	3.85	3.64	3.53	4.11	3.85	3.44
**COL_PUB** (Colleague Publications)	2.75	2.69	2.83	2.44	2.52	2.61	2.94	2.90	3.09	2.55	2.81	2.71
**SHR_TAT** (Short Turnaround Time)	3.37	3.28	3.46	3.31	3.02	3.59	3.35	3.68	3.50	3.58	3.44	2.99
**HYB_APC** (Hybrid APC Option)	2.78	2.76	2.81	2.53	2.82	2.67	2.63	3.21	2.76	2.73	2.88	2.66
**ACC_RATE** (Acceptance Rate)	3.01	2.93	3.09	3.15	2.62	3.83	2.97	3.10	3.35	3.35	2.92	2.69

Gender once again emerged as a robust explanatory factor here. Using the Student’s *t*-test for independent samples, significant differences were identified based on gender. Women assigned higher priority to global visibility and free access. This divergence was particularly profound regarding interest in full Open Access (DIA_OA), which recorded the highest significance and the largest effect size ( *p* < .001; *d* = −0.37). Furthermore, gender notably influenced the importance attributed to a journal’s reputation (REP_BRD: *p* = .010; *d* = −0.29) and to its findings being read by a wider audience (WID_AUD: *p* = .003; *d* = −0.33); in all cases, female researchers demonstrated a stronger support in this regard.

Regarding variations according to the research career stage (R1–R4), the analysis of variance (ANOVA) revealed that publication priorities evolve as researchers become more established within the system. The importance assigned to the acceptance rate (ACC_RATE) presented highly significant differences ( *F* (3, 326) = 6.07; *p* < .001; η ^2^ = .053). Early-career profiles (R1 and R2, both *M* = 3.35) prioritised securing publication, compared to a lower level of concern among the R4 group ( *M* = 2.69). Interest in full Open Access without costs (DIA_OA) peaked during the R2 stage ( *M* = 4.11), differing significantly ( *p* = .001; η ^2^ = .047) from the two extremes of the professional career (R1: *M* = 3.53; R4: *M* = 3.44). Meanwhile, urgency for short decision timeframes (SHR_TAT) ( *p* = .004; η ^2^ = .041) was a critical priority for R1 ( *M* = 3.50) and R2 ( *M* = 3.58), but lost relative weight in the senior R4 stages ( *M* = 2.99).

Finally, the field of research acts as the most influential factor in shaping publication strategies. While reputation and impact factor were shared values ( *p* > .05), specific disciplinary patterns were observed in our study. Health Sciences and Arts and Humanities stood out for a significantly higher evaluation of free Open Access (DIA_OA: *p* < .001; η ^2^ = .062), reaching a peak in Arts and Humanities ( *M* = 4.21). Similarly, Health Sciences led the concern regarding acceptance rates (ACC_RATE: *M* = 3.83). Engineering and Architecture was the area that most valued editorial decision speed (SHR_TAT: *M* = 3.68; *p* = .009) and showed the greatest predisposition towards Open Access options involving APC payments (HYB_APC: *M* = 3.20; *p* = .009), in stark contrast to Arts and Humanities ( *M* = 2.52). Researchers in the Sciences gave, in comparative terms, the lowest evaluation to both acceptance rates ( *M* = 2.62) and full Open Access ( *M* = 3.32), suggesting an approach more focused on traditional publication channels.

### Evolution of knowledge regarding open science instruments (2021–2024): oa routes, repositories, and data management

**Figure 1.**
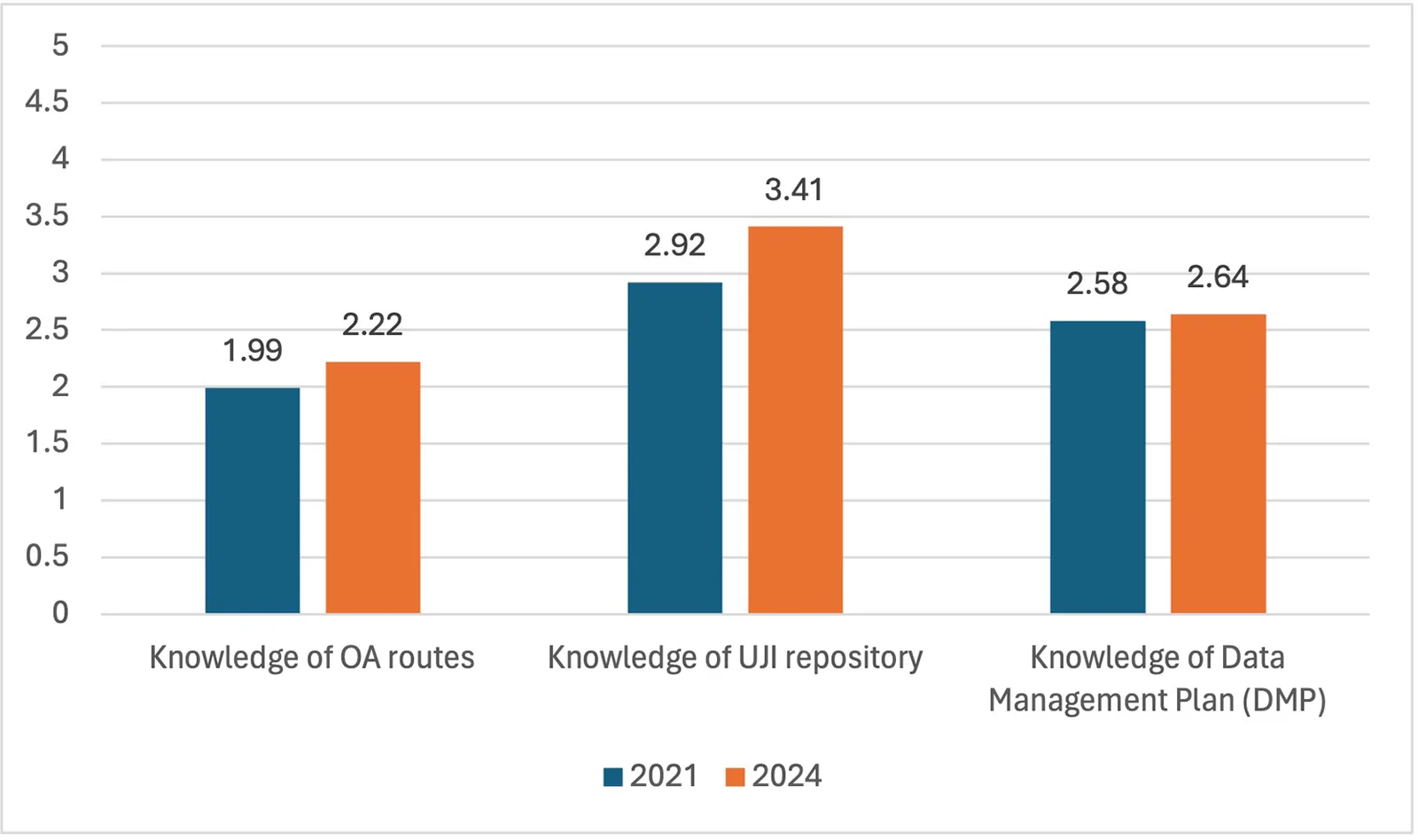
Knowledge of Open Science Resources: Comparison by Year (2021 vs. 2024). Mean Values on a 1–5 Scale. **Source:** Authors’ own work. **Note**: Values represent the means (M) obtained on a Likert scale from 1 (None) to 5 (Expert). Significant increases are observed across all dimensions, with the Institutional Repository standing out as the tool with the highest degree of knowledge and growth ( *p* < .001).

The support for Open Access (OA) observed in the previous section leads to the question of whether there is a corresponding mastery of its various procedural aspects. The comparative analysis between 2021 and 2024 reveals a positive, albeit modest, advancement in researcher literacy. As Figure 1 shows, self-perceived knowledge increased across all analysed dimensions, suggesting a slow but progressive improvement. However, this growth still leaves most indicators in the lower range of the scale, indicating that practical mastery is currently far from expert levels.

Despite being the dimension with the lowest score, Knowledge of Open Access routes displayed an upward trend, rising from *M* = 1.98 to *M* = 2.21. The Institutional Repository (UJI) was the instrument with the greatest growth in knowledge, evolving from *M* = 2.92 in 2021 to M = 3.41 in 2024, exceeding the theoretical “pass” threshold for the first time. Knowledge regarding Data Management Plans (DMPs) displayed relative stability, with only a slight increase in scores (from *M* = 2.57 in 2021 to *M* = 2.64 in 2024). It is worth noting that, in both editions, scores for Knowledge of Data Management Plans remained higher than those of OA Routes.

The comparison of means for independent samples revealed no significant differences by gender. Furthermore, the one-way analysis of variance (ANOVA) yielded no statistically significant differences by field of knowledge. However, differences were observed for the research stage. Thus, the research career stage (R1–R4) was confirmed as the primary factor affecting knowledge.

In 2021 (N = 505), academic seniority acted as a driver for literacy. Knowledge of the UJI Repository showed a linear increase from R1 profiles ( *M* = 2.43) to R4 profiles ( *M* = 3.18), with very significant differences ( *F* (3, 501) = 8.79; *p* < .001; η ^2^ = .050). Similarly, Knowledge of DMPs was higher among senior profiles ( *M* = 2.78) than early-career researchers ( *M* = 2.17; *p* = .001; η ^2^ = .032; see Table 9).

**Table 9.** Knowledge of open science resources. 2021. Means by gender, research area and career stage.

	Mean	Men	Women	AH	S	HS	SLC	EA	R1	R2	R3	R4
**ROUTE** (Routes Knowledge)	1.99	1.98	1.99	1.97	2.01	2.16	2.01	1.91	1.75	2.03	1.96	2.16
**REP** (Repository Knowledge)	2.92	2.92	2.93	2.81	2.87	2.92	2.96	2.96	2.43	2.89	2.93	3.18
**DMP** (DMP Knowledge)	2.58	2.58	2.58	2.45	2.60	2.80	2.58	2.57	2.18	2.56	2.58	2.79

Professional category remained a decisive factor in 2024 (N = 330), three years on from the first survey, although a trend towards homogenisation was observed in certain instruments. The UJI Repository continued to be the variable with the widest professional gap ( *F* (3, 326) = 7.82; *p* < .001), with a robust effect size (η ^2^ = .067). Regarding knowledge of Data Management Plans, the gap detected in 2021 was less clear, reaching borderline significance ( *p* = .061). This suggests that knowledge of data management is becoming more uniform (albeit moderate) across all career levels. However, Open Access Routes displayed no differences in relation to knowledge by category ( *p* = .152). This result, supported by a minimal effect size (η ^2^ = .017), confirms that understanding publication routes is no longer acquired through “years of experience”; rather, it appears to depend on other cross-cutting factors, such as specific training or exposure to new dissemination policies ( Table 10).

**Table 10.** Knowledge of open science resources. 2024. Means by gender, research area and career stage.

	Mean	Men	Women	AH	S	HS	SLC	EA	R1	R2	R3	R4
**ROUTE** (Routes Knowledge)	2.22	2.21	2.22	2.25	2.09	2.76	2.19	2.22	1.88	2.30	2.18	2.35
**REP** (Repository Knowledge)	3.41	3.45	3.37	3.48	3.26	3.17	3.48	3.44	2.79	3.32	3.50	3.61
**DMP** (DMP Knowledge)	2.64	2.71	2.58	2.33	2.68	2.83	2.65	2.78	2.38	2.48	2.67	2.86

### Knowledge of specific open access publication routes and preprint/postprint versions (2024)

**Figure 2.**
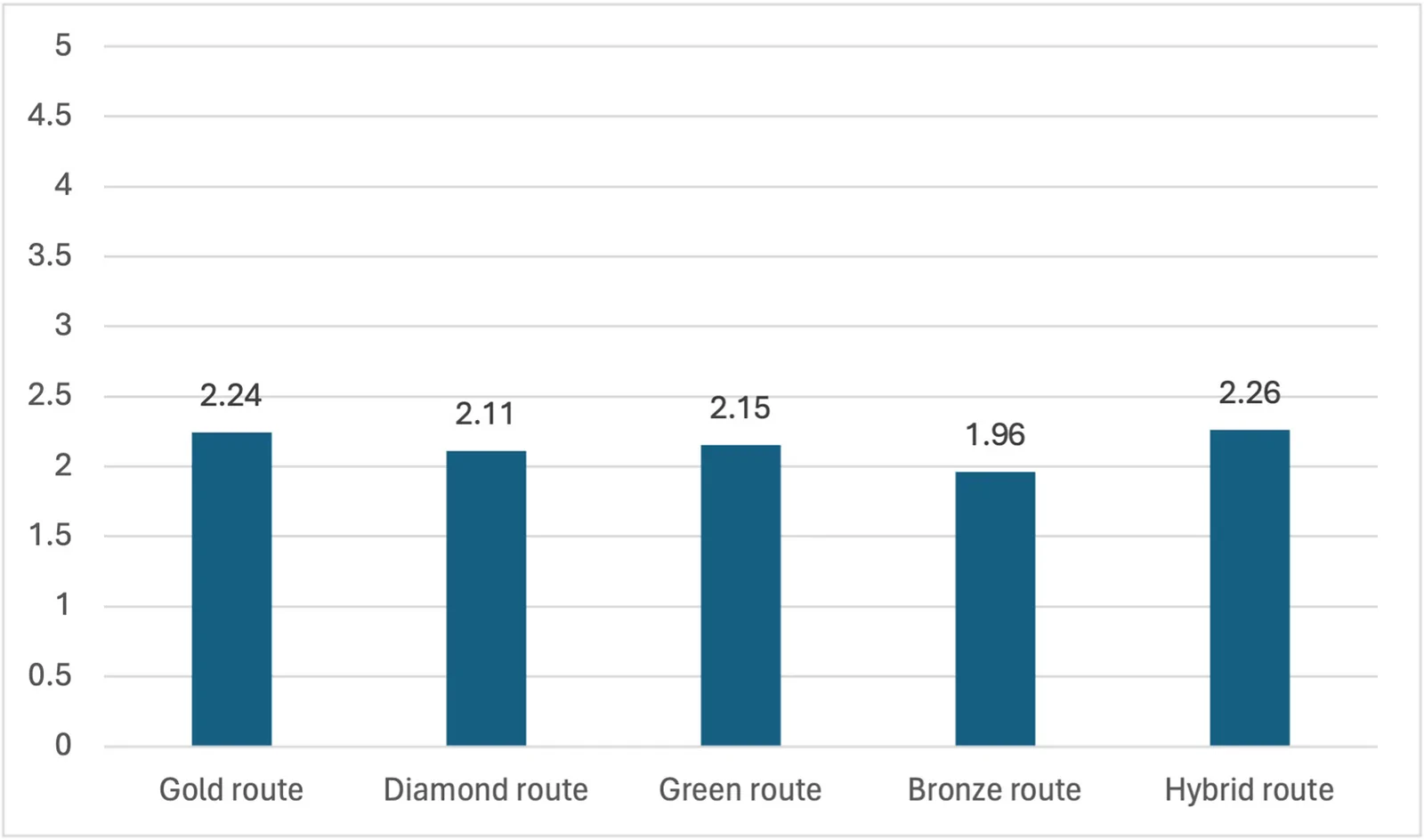
Self-reported Knowledge of Open Access Publishing Routes. 2024. **Source:** Authors’ own work. **Note:** Means correspond to a Likert scale from 1 (None) to 5 (Expert).

The 2024 edition delved deeper into the research community’s declared knowledge of Open Access by enquiring about specific understanding of OA publication routes and the management of manuscript versions (preprints and postprints). This information represents a step forward compared to the findings of 2021. Through these questions, it was established that the university community reports a notably low self-perceived competence in distinguishing between different publication routes (see Figure 2). With regard to the article itself, the format of the main body is flexible: it should be concise, making it easy to read and review, and presented in a format that is appropriate for the type of study presented.

On a scale from 1 to 5, all dimensions fell below the theoretical midpoint of knowledge (3.00), highlighting a profound gap in terms of training. The Hybrid route ( *M* = 2.26; *SD* = 1.19) and Gold route ( *M* = 2.24; *SD* = 1.20) were the dimensions with the highest degree of familiarity, though levels remained low. The Diamond and Green routes displayed a pronounced positive skew (0.65 to 0.93), confirming that most responses were concentrated at the lowest values on the scale. In other words, the majority of researchers acknowledged an almost complete lack of knowledge of both routes. Finally, the Bronze route ( *M* = 1.96; *SD* = 0.99) displayed the most deficient level of knowledge and the greatest consensus in this regard (lowest dispersion). The Student’s t-test for independent samples yielded no significant differences by gender. Furthermore, ANOVA detected no significant differences by field or category across these specific variables ( Table 11).

**Table 11.** Self-reported knowledge of specific open access publishing routes. 2024. Means by gender, research field and professional category.

	Mean	Men	Women	AH	S	HS	SLC	EA	R1	R2	R3	R4
**GOLD** (Gold)	2.24	2.34	2.13	2.10	2.06	2.50	2.27	2.38	2.12	2.19	2.21	2.41
**DIA** (Diamond)	2.11	2.12	2.10	2.21	1.92	2.50	2.17	2.00	1.79	2.15	2.08	2.27
**GRE** (Green)	2.15	2.17	2.13	2.08	1.97	2.78	2.16	2.17	1.94	2.12	2.11	2.33
**BRO** (Bronze)	1.96	1.96	1.96	1.98	1.82	2.50	1.98	1.90	1.88	1.93	1.93	2.08
**HY** (Hybrid)	2.26	2.24	2.29	2.02	2.08	3.17	2.25	2.39	1.94	2.22	2.22	2.52

In contrast to Publication Routes, Knowledge regarding Manuscript Versions (preprint/postprint) displayed a more robust mean score ( *M* = 3.50). However, this literacy was heavily conditioned by discipline and professional stage. In this regard, highly significant differences were found ( *F* (4, 325) = 5.01; *p* < .001) according to field of knowledge, with discipline being the factor with the greatest explanatory weight (η ^2^ = .058). Specifically, Health Sciences ( *M* = 3.83) and Sciences ( *M* = 3.79) obtained the highest scores in terms of knowledge, which reflects their longer-standing use of repositories. Conversely, Arts and Humanities ( *M* = 3.32) and Social and Legal Sciences ( *M* = 3.24) presented the largest knowledge gaps in this regard ( Table 12). This latter finding is critical, given that these disciplines represented the highest volume of participation in the sample ( *n* = 125).

**Table 12.** Self-reported knowledge of preprint/postprint. 2024. Means by gender, research field and professional category.

	Mean	Men	Women	AH	S	HS	SLC	EA	R1	R2	R3	R4
**PRE/POST** (Preprint/Postprint Knowledge)	3.50	3.58	3.41	3.33	3.79	3.83	3.25	3.71	3.06	3.40	3.54	3.71

Career progression also displayed real differences ( *p* = .014; η ^2^ = .032), although with half the explanatory power of field of knowledge. The lowest level of knowledge was found in the R1 professional category ( *M* = 3.06), increasing notably in R2 ( *M* = 3.40) and R3 ( *M* = 3.54), before reaching its peak in R4 ( *M* = 3.71). This confirms that, in the management of manuscript versions, thematic specialisation is a more powerful predictor than academic rank.

### From data to voices: Qualitative comments in the survey (2024)

The comments provided in the survey, while not numerous, served as an explanatory reinforcement of the findings analysed in the quantitative section. These testimonies revealed a certain tension: while, on the one hand, a degree of ignorance remained regarding the implications of Open Access, on the other, the research community was particularly critical of the publishing industry.

Consequently, there was an explicit rejection of the Article Processing Charge (APC) model, which researchers perceive as a process that accentuates the mercantilisation of scientific publishing. This stance was evident in statements describing the model as a “scam that enriches the publisher” (F.HS.R2) or denouncing the inequity it generates: “Disseminating results should not cost us money; it creates categories within each field, as money provides access to publishers that are higher in the rankings” (F.AH.R3). Thus, the critical position towards monetisation within the Arts and Humanities community—previously observed in the quantitative data—was ratified by testimonies describing the act of paying to publish as “shameful” (M.AH.R3). This view was shared by researchers in other fields, who pointed out the scale of these costs, which account for “a high percentage of the execution costs of our publicly funded research projects” (M.S.R3). Even among early-career profiles, frustration towards commercial publishers reached critical levels, with some advocating for radical measures due to the lack of social return on public investment: “I think all scientific publishers should be expropriated; they take everything we write […] without contributing a single penny” (M.EA.R1).

Furthermore, the qualitative quotes help explain why the Impact Factor remained the primary criterion for journal selection despite personal convictions. A senior researcher summarised this systemic contradiction: “High impact factor and short decision timeframes should not be the main factors. But currently they are, because of the way productivity in science is measured” (F.EA.R3).

Finally, the low levels of technical knowledge detected regarding specific Open Access routes ( Figure 2) were reflected in a sense of general confusion. Some respondents acknowledged that, despite their seniority, they felt they had “limited knowledge based on experience and hearsay”, but they “truly didn’t end up understanding anything at all” (M.S.R4). In other cases, the lack of familiarity was linked to an ignorance of prestigious fee-free alternatives, with one respondent noting: “I do not know of any prestigious journal in my field that is cost-free for both readers and authors simultaneously” (M.EA.R4).

## Discussion

The results obtained in this study confirm that researchers at the Universitat Jaume I (UJI) maintain a broadly favourable stance towards Open Access (OA), a trend that has not only consolidated but intensified. This near-unanimous consensus on fundamental principles—such as the conception of science as a non-monetisable common good or the right to free reading—aligns the UJI with findings reported in the literature ( Dallmeier-Tiessen et al., 2011; Serrano-Vicente et al., 2016; Zhu, 2017; Segado-Boj et al., 2018). In the specific case of our work, it is particularly revealing that “Open Access” status has gained in importance as a journal selection criterion. This finding is novel; while previous literature positioned it as a peripheral factor with limited influence on decision-making ( McDonald et al., 2016; Narayan et al., 2018), at UJI it emerges as a truly decisive factor. This shift suggests a local paradigm change, likely driven by recent reforms in Spanish evaluation systems (ANECA), which have begun to reward openness, allowing researchers to align the pursuit of academic prestige with open publishing without the ethical or economic conflicts of previous years.

However, this support is neither monolithic nor uniform. Gender emerges as a critical moderating variable in this respect, revealing significant gaps in researchers’ perceptions regarding Open Science. Specifically, stronger support is observed among female researchers regarding the consideration of research as a non-monetisable common good and its capacity to drive innovation (p < .001). These results point in the same direction as those observed by ( Kaba & Said, 2015) who, in the context of Al Ain University, highlighted that female researchers have significantly greater awareness and more positive perceptions regarding the utility of open resources than their male colleagues (p < 0.05), while also registering a higher frequency of use of these materials among women (an average of 8.42 resources compared to 5.29 for men).

As our results show, differences in perceptions of OA are also accentuated by field of research. This finding confirms that attitudes in this respect are mediated by disciplinary “publication cultures” ( McDonald et al., 2016), where areas such as Arts and Humanities or Health Sciences lead the rejection of monetisation when compared with the more pragmatic views held in the Sciences or Engineering. Notably, in Health Sciences, full support is found for free reading, reflecting an awareness of the social impact of medical knowledge that exceeds means observed in other contexts ( Boock et al., 2020). Furthermore, professional trajectory emerges as a critical factor that breaks the idea of a linear commitment. The “peak” of support in mid-career profiles (R2 and R3) suggests that conviction regarding OA reaches maturity once researchers have moved beyond initial precariousness but have not yet adopted the potential scepticism of senior stages ( Segado-Boj et al., 2018). Conversely, early-career researchers (R1) present an interesting paradox: although they are “digital natives” in their information consumption, they show less rejection of the system being monetised. Following Serrano-Vicente et al. (2016), this could be explained by strong competitive pressure and the need for survival in the current accreditation system, forcing them to subordinate their ideals to the demands of publishing in high-impact journals, often under closed or hybrid models.

Regarding literacy and self-reported knowledge of Open Access, the data reveal the gap that gives this study its title: “academics hear and like the music, but do not know the lyrics”. This distance echoes previous research such as the SOAP project ( Dallmeier-Tiessen et al., 2011), where a notable gap was observed between 89% of support for OA and a mere 8% of actual publication using this route. Our results suggest that this disparity between attitude and practice could be rooted in a lack of procedural literacy. Thus, despite high support for the general concept—in line with studies in Spain, the United States, or Bulgaria ( Ruiz-Pérez & Delgado-López-Cózar, 2017; Yang & Li, 2015; Boock et al., 2020)—an alarming lack of specificity regarding publication routes persists. The declared ignorance of the Diamond and Green routes is notable. However, this deficit is not exclusive to the UJI research community but aligns with a global trend of “illiteracy” observed in Canada, Australia, and Israel ( McDonald et al., 2016; Narayan et al., 2018; Aharony & Hadad, 2022). As other studies have shown, the disconnection is especially acute when enquiring about milestones such as the Budapest Declaration or tools like Sherpa/Romeo (now part of Jisc Open Policy Finder) or Plan S, confirming that most authors are unfamiliar with the “rules of the game” or the infrastructures that guarantee their sovereignty over scientific production ( Hernández-Borges et al., 2006; Taylor & Francis, 2019).

In the face of this deficiency, the institutional repository emerges as the “success story” of our study. While international literature reports warning figures regarding ignorance or fear of using repositories due to copyright conflicts ( Yang & Li, 2015; Sheikh, 2019; Aharony & Hadad, 2022), UJI staff have managed to normalise its use, exceeding the “pass” mark. This progress is particularly visible in the mastery of preprint and postprint concepts, essential tools for the Green route. Nevertheless, it is important to note that this momentum has not spread uniformly across the campus, a literacy gap being identified in which academic discipline acts as the primary differentiating factor, even above research experience. On the other hand, this positive evolution cannot be understood without considering the role of institutional training and, decisively, regulatory changes in Spain. The reform of the *sexenios* (six-year research periods) in December 2023, which includes the promotion of Open Access in its criteria, has acted as a catalyst for greater awareness.

In conclusion, the results of this study suggest that UJI is in a position where the “music” of Open Access is heard with increasing clarity and enjoys widespread adherence and support. However, the findings also indicate that a greater literacy regarding publication routes and archiving dynamics is still required.

## Study limitations

Despite the contributions of this study to understanding the evolution of OA in the academic environment, certain limitations that define the scope of the results must be acknowledged. Firstly, the research is confined to a single institution, the Universitat Jaume I. While this case-study approach allows for an in-depth and contextualised analysis of the response to specific policies, the results are not automatically generalisable to the entire Spanish university system. Similarly, although the 2021–2024 period captures a cycle of intense regulatory changes, the study would benefit from a broader longitudinal framework. A greater temporal span would allow for observations on whether practical knowledge of Open Access eventually becomes structurally integrated into research culture.

Secondly, it should be noted that the study is based on researchers’ self-perceived knowledge. Measuring the literacy of an academic community is complex, and this work does not utilise performance tests but rather self-assessment. Nevertheless, the consistency and sincerity of the responses should be highlighted: the data show no signs of social desirability bias or exaggerated values. On the contrary, the tendency to report limited practical knowledge, even after training periods, reinforces the validity of the results and suggests that researchers are acutely aware of their own procedural gaps.

Finally, it should be noted that this analysis is predominantly quantitative, given that it is based on surveys. Future approaches could benefit from methodological triangulation, incorporating qualitative techniques (such as in-depth interviews or focus groups to explore the underlying factors behind barriers to acquiring procedural literacy). Furthermore, expanding the sample to other national and international contexts would help confirm whether the trends observed at the Universitat Jaume I reflect a more widespread lack of literacy in the transition to Open Access. Despite these limitations, this approach offers a solid basis for analysing an ecosystem shaped by active Open Access policies, ensuring the high relevance of these findings.

## Conclusions

While progress in consolidating Open Access at the Universitat Jaume I appears to be advancing, actual mastery of its implications is moving at a significantly slower pace. A persistent gap is confirmed between the acceptance of the “music” (the ideal of science as a common good) and knowledge of the “lyrics” (the routes and management mechanisms). This suggests that Open Access literacy involves a complex learning process that does not occur spontaneously simply through the act of researching and publishing.

This study also demonstrates that commitment to Open Access is mediated by gender, academic career stage, and field of knowledge, and that the explanatory power of these factors has evolved over time. Furthermore, it highlights that institutional policies and the reform of evaluation systems—such as the recent update to the *sexenios* criteria in Spain—appear to act as fundamental drivers of change, although their integration into academic culture is an intrinsically gradual and slow process. In this regard, the observed improvement in the knowledge and use of the institutional repository shows that progress can be achieved when evaluation incentives are aligned with a consistent training. However, this study also suggests that only sustained efforts in training and science policy will fully integrate Open Access into research practice.

## Ethics and consent

Formal ethical approval for this research was obtained from the Ethics Committee for Research Involving Human Beings (Comité de Ética en la Investigación con Seres Humanos, CEISH) at the Universitat Jaume I (Castellón, Spain). The study was conducted in two phases under the following approvals:

Phase 1 (2021 Survey): Formal ethical approval granted in April 2021 (Reference No. CD/45/202).

Phase 2 (2024 Survey): Formal ethical approval granted in July 2024 (Reference No. CEISH/58/2024).

The committee’s reports confirm that all procedures meet the required ethical standards for research involving human participants. As detailed in the Methods section, explicit electronic informed consent was obtained from all respondents prior to participation, ensuring full compliance with the GDPR and current national legislation.

## Declaration of generative AI and AI-assisted technologies

During the preparation of this manuscript, the authors used Gemini (version June 2026) to assist with English language polishing and to refine the writing. After using this tool, a native-speaking editor proofread the draft, and the authors reviewed and edited the content as needed. The authors take full responsibility for the final text and the accuracy of the manuscript.

## Data Availability

This project contains the following underlying data, hosted at the Universitat Jaume I Repository. The datasets include the raw survey responses from the 2021 and 2024 editions regarding Open Access perceptions and practices.
•
**Data file 1:** Survey Data (2021 Edition). Permanent URI/Handle: http://hdl.handle.net/10234/203683 ( Feenstra et al., 2023). •
**Data file 2:** Survey Data (2024 Edition). Permanent URI/Handle: http://hdl.handle.net/10234/734895 ( Mora & Feenstra, 2025). This dataset contains the anonymised survey responses used as the baseline for the comparative analysis. To ensure strict participant anonymity and maintain full compliance with GDPR guidelines, the gender variable was excluded from the shared dataset. Given the low representation of female researchers in specific academic disciplines, particularly at senior career stages (R4), cross-referencing gender with discipline and rank presented a severe risk of deductive re-identification. Consequently, a privacy-by-design approach was prioritised over demographic breakdown to safeguard the identities of individuals in minority groups. Data are available under the terms of the Creative Commons Attribution 4.0 International license (CC BY 4.0). The questionnaire tools used for this data collection are also openly available within the respective datasets.
•
**File 1:** Question Flow Survey 2021. Permanent URI/Handle: http://hdl.handle.net/10234/203683 ( Feenstra et al., 2023). •
**File 2:** Question Flow Survey 2024. Permanent URI/Handle: http://hdl.handle.net/10234/734895 ( Mora & Feenstra, 2025). Extended data are available under the terms of the Creative Commons Attribution 4.0 International license (CC BY 4.0).

## Footnotes

^1^

https://www.ciencia.gob.es/Estrategias-y-Planes/Estrategias/ENCA.html

^2^

https://www.uji.es/investigacio/base/etica/cbpid/

^3^

https://www.uji.es/estudis/centres/escola-doctorat/base/arxiu/Formacio-transversal/25-26/organitzacio/ and https://www.uji.es/estudis/centres/escola-doctorat/base/arxiu/Formacio-transversal/25-26/practiques/

^4^

https://www.uji.es/investigacio/base/enca/noticies/termometre/informe_ang.pdf

^5^

https://www.uji.es/upo/rest/contenido/1039818540/raw?idioma=es
