# Supplementary material for: Open Access: hearing the music but not knowing the lyrics. The evolution of knowledge and perceptions among researchers at a Spanish university (2021–2024)
Source: Open Res Eur. 2026 Jul 6;6:236. [Version 1] doi: 10.12688/openreseurope.24242.1 (PMC13560693; doi:10.12688/openreseurope.24242.1)
Supplement: Peer review history

# Reviewer report: Reviewer response for version 1

**DOI:** 10.21956/openreseurope.26250.r77000

**Published:** 2026-08-08

**Authors:** Abdelghani Maddi

**Affiliations:** 1 CNRS, Paris, France

This paper compares researchers’ attitudes towards and self-reported knowledge of Open Access at Universitat Jaume I in 2021 and 2024. The topic is relevant, particularly given the institutional and regulatory changes that occurred in Spain during this period. The manuscript is generally clear, the response rates are substantial for an institutional survey, and the availability of the questionnaires and anonymised datasets is a clear strength.

 The main contribution of the paper lies, in my view, in documenting the persistent gap between researchers’ broad support for Open Access and their much more limited familiarity with its practical mechanisms. The results concerning the institutional repository, data management plans, and the different OA publishing routes are potentially useful for universities designing training and support policies.

 However, the manuscript currently goes beyond what the design and data allow. I have several reservations about the way the comparison between 2021 and 2024 is currently framed. Most of them concern the limits of the research design and the interpretation of the statistical results. I believe these issues can be addressed through a substantial revision, without changing the main purpose of the paper.

**Major comments**

**1. The design does not support causal claims about institutional policies**

 The paper is based on two independent cross-sectional surveys, not on a panel following the same individuals. The authors should describe it consistently as a repeated cross-sectional study.

 The two surveys provide useful snapshots of the institution in 2021 and 2024, but they do not allow the authors to establish that institutional training, national reforms, ANECA changes or the *sexenios* caused the observed differences. Changes may also reflect differences in respondents, non-response patterns, or broader developments in the Spanish research system.

 This point is particularly important because the professional composition of the samples was not fully representative, while professional category is subsequently associated with several outcomes.

 Claims about the “effectiveness” of policies, “drivers of change” or a “catalyst” effect should therefore be revised throughout the abstract, discussion and conclusion. These factors may be discussed as plausible explanations, but not as demonstrated effects.

 The abstract should also be more cautious. The study provides evidence on changes observed within one institution, rather than direct evidence of the effectiveness of Open Science policies.

**2. Only a limited number of variables are directly comparable across years**

 The manuscript usefully explains that the 2024 questionnaire was expanded and that some response formats changed. Only a small number of items appear to be strictly identical across the two surveys: knowledge of OA routes, awareness of the institutional repository, and knowledge of Data Management Plans.

 The comparison of publication criteria is much weaker. In 2021, respondents selected factors from a binary list. In 2024, they rated partly different factors on a five-point scale. In addition, “Open Access availability” in 2021 is compared with a specific “Diamond Open Access” item in 2024.

 The comparison between 34.5% in 2021 and a mean of 3.78 in 2024 is therefore not a valid measure of change. These results may be presented as two separate descriptions of publication priorities, but they do not show that Open Access became more important over time.

 I suggest distinguishing clearly between:

- identical items that can be compared directly;
- new variables measured only in 2024;
- publication criteria that can only be discussed descriptively within each survey.

The statement that the institution experienced a “local paradigm change” should either be removed or substantially qualified.

**3. The statistical analysis needs clarification**

 The Methods section describes gender comparisons using t-tests and comparisons by field and professional category using ANOVA. It does not clearly describe how the authors tested differences between 2021 and 2024, although Figure 1 states that significant increases occurred across all three common indicators.

 For the identical items, the authors should report direct comparisons between the two independent samples, including:

- the difference in means;
- a confidence interval;
- the test used;
- an effect size;
- the number of valid observations.

An adjusted regression including year, field and professional category would also help determine whether the observed changes remain after accounting for differences in sample composition.

 This is especially important for DMP knowledge, where the difference between the two years is very small. Statistical significance, if present, should not be confused with practical importance.

 The paper also reports many t-tests and ANOVAs. The authors should explain whether the analyses are exploratory and how they addressed multiple testing. Several conclusions rely on p-values close to .05, which should be interpreted cautiously.

 The following points also require clarification:

- treatment of missing values;
- assumptions of t-tests and ANOVAs;
- post-hoc tests used after significant ANOVAs;
- adjustment of pairwise comparisons;
- confidence intervals for effect sizes.

There also appears to be an error in the 2021 gender comparison: the manuscript reports (t(517)), although the total sample is 505.

 Finally, terms such as “explanatory factor”, “moderating variable” and “predictor” are too strong for separate bivariate analyses. Unless multivariable models are added, the paper should simply state that outcomes differed by gender, field or career stage.

**4. Some conclusions do not correspond exactly to the measures**

 The study measures self-reported knowledge and awareness, not objectively assessed competence. The manuscript should therefore avoid moving too quickly from self-assessment to “mastery”, “literacy” or “actual knowledge”.

 The repository item measures awareness of the repository and its possibilities. It does not measure actual use, deposit behaviour or correct understanding. The conclusion that repository use has been “normalised” is therefore not directly supported.

 Similarly, the midpoint of a Likert scale should not be described as a “pass threshold” unless this interpretation was defined in advance and justified.

 The DAP item also needs reinterpretation. Respondents were asked whether access to data was more important than access to articles. A mean around the midpoint does not indicate weak support for open data; it may simply mean that respondents do not consider one more important than the other.

 The claim that the data show no evidence of social-desirability bias should be removed. No measure of this bias was included.

 The classification of Gold, Green, Bronze, Hybrid and Diamond as equivalent “routes” should also be explained more carefully. These categories refer to different dimensions and may overlap. The paper should specify what definitions were given to respondents. Without definitions, the results mainly measure familiarity with labels.

**5. The qualitative analysis and reproducibility need more detail**

 The comments are useful, particularly those concerning APCs and the tension between researchers’ values and evaluation incentives. However, the paper refers to a thematic analysis without explaining:

- how many respondents provided comments;
- how the comments were coded;
- whether coding was inductive or predefined;
- how quotations were selected;
- whether more than one researcher participated in the analysis.

In its current form, the section reads more like a selection of illustrative comments. The authors should either provide the missing methodological detail or describe the section more modestly as illustrative qualitative comments.

 There is also a possible anonymity issue. Quotations are labelled by gender, field and career stage, while the authors explain elsewhere that this combination creates a risk of re-identification. The quotation labels should be simplified.

**Minor comments**

- Use consistent numerical values and rounding throughout. Some means differ slightly between the text, figures and tables.
- Report standard deviations or confidence intervals alongside means.
- Two decimal places are sufficient; four decimals in Table 7 are unnecessary.
- Give the sample size for each subgroup, especially for Health Sciences.
- Clarify what constitutes a “valid response” and how incomplete questionnaires were treated.
- Remove the sentence on page 12 beginning “With regard to the article itself…”, which appears to be residual template text.
- Correct “Student’st-test”.
- Replace “practical or clinical relevance” with “practical relevance”.
- Check the bibliographic details of the Aharony and Hadad reference.

To sum up, the paper addresses a relevant question and contains useful empirical material. Its main finding (i.e., the contrast between broad support for Open Access and limited self-reported knowledge of its practical mechanisms) is supported by the data.

 The manuscript would be stronger if the authors limited direct temporal conclusions to identical items, reported formal year comparisons, accounted more carefully for sample composition, and avoided attributing the observed differences to institutional policies without an appropriate causal design.

 I recommend indexing after these issues have been addressed.

If applicable, is the statistical analysis and its interpretation appropriate?

Partly

Is the study design appropriate and is the work technically sound?

Partly

Is the work clearly and accurately presented and does it engage with the current literature?

Yes

Are the conclusions drawn adequately supported by the results?

Partly

Are sufficient details of methods and analysis provided to allow replication by others?

Partly

Are all the source data and materials underlying the results available?

Yes

Reviewer Expertise:

Scientometrics, Open Science, Research Integrity, Research Evaluation

I confirm that I have read this submission and believe that I have an appropriate level of expertise to confirm that it is of an acceptable scientific standard, however I have significant reservations, as outlined above.

# Reviewer report: Reviewer response for version 1

**DOI:** 10.21956/openreseurope.26250.r77004

**Published:** 2026-07-22

**Authors:** Remedios Melero

**Affiliations:** 1 Spanish National Research Council (CSIC), Burjassot, Valencian Community, Spain

The paper is in English; however, the survey text and the linked dataset are in two languages other than that of the article, which makes it difficult for the reader to follow. The authors should produce English versions.

 The dataset is not coded by scores; the questions are jumbled together and are not easy to interpret. It is suggested that a version be produced organised into blocks, with the survey ratings presented numerically; otherwise, in order to reuse it, a significant amount of cleaning and adaptation work will be required.

 The 2024 survey includes questions supposedly about open science, but most focus on open access; however, they do include a section on data management, which would be more appropriately categorised under ‘data’. I have noticed that the survey includes questions that are indeed related to open science, such as open peer review, or awareness of DORA or CoARA. I believe the authors should have used all this data to analyse different groups based on responses and variables such as gender and academic position.

 In the Methodology section, the authors should provide the full list of survey items included in the five blocks; a table would suffice.

 There are sections in the methodology that relate to results, for example:

*976 members of the PDI/PI, yielding 330 valid responses (33.81%), with a sampling error of 4.39%. The slightly lower population figure compared to 2021 is due to a reduced presence of research staff and certain scholarship programmes in the 2024 institutional census.*

 The authors should review this entire section.

 Was the survey anonymous? If so, no personal data was requested; gender is not a variable that needs to be removed from the responses, as it is not a unique identifier. OR did you pseudo-anonymise the responses?

 About the 2024 survey, did you determine the Cronbach's alpha (α) to evaluate the internal consistency of the survey results?

 Did you publish the results of 2021 survey before?

 Sometimes you include gender as variable but then you remove it:

*Gender once again emerged as a robust explanatory factor here. Using the Student’s t-test for independent samples, significant differences were identified based on gender. Women assigned higher priority to global visibility and free*

*access. This divergence was particularly profound regarding interest in full Open Access (DIA_OA), which recorded thehighest significance and the largest effect size (p < .001; d =0.37). Furthermore, gender notably influenced the*

*importance attributed to a journal’s reputation (REP_BRD: p = .010; d =*

*0.29) and to its findings being read by a wider audience (WID_AUD: p = .003; d =*

*0.33); in all cases, female researchers demonstrated a stronger support in this*

 Results are presented within the text, it would be more convenient to tabulate them and give the statistics as part of te tables

 Page 10. Why did you not separate versions preprints/postprints, results could have been different:

*In contrast to Publication Routes, Knowledge regarding Manuscript Versions (preprint/postprint) displayed*

*p. 14. It says:*

*Regarding literacy and self-reported knowledge of Open Access, the data reveal the gap that gives this study its title:“academics hear and like the music, but do not know the lyrics”. This distance echoes previous research such as the SOAP*

*project (Dallmeier-Tiessen et al., 2011), where a notable gap was observed between 89% of support for OA and a mere 8% of actual publication using this route.*

 This reference is too old for this theme; there is more recent references regarding this subject.

 The university has an open access policy; the authors could have studied the effect of it from 2021 to 2024.

If applicable, is the statistical analysis and its interpretation appropriate?

Partly

Is the study design appropriate and is the work technically sound?

Partly

Is the work clearly and accurately presented and does it engage with the current literature?

Partly

Are the conclusions drawn adequately supported by the results?

Partly

Are sufficient details of methods and analysis provided to allow replication by others?

Partly

Are all the source data and materials underlying the results available?

Yes

Reviewer Expertise:

Open access, open science open data

I confirm that I have read this submission and believe that I have an appropriate level of expertise to confirm that it is of an acceptable scientific standard, however I have significant reservations, as outlined above.

# Reviewer report: Reviewer response for version 1

**DOI:** 10.21956/openreseurope.26250.r77007

**Published:** 2026-07-21

**Authors:** Altaf Ali

**Affiliations:** 1 Aligarh Muslim University, Aligarh, Uttar Pradesh, India

The study entitled “Open Access: hearing the music but not knowing the lyrics. The evolution of knowledge and perceptions among researchers at a Spanish university (2021–2024) is a great work. The paper covered the timely topic and has a great importance to contribute to existing literature. Whole the manuscript is well organised.

  

1. **Introduction**: In the first paragraph of the Introduction section, the authors mention the initiative “Plan S”. To give a wider context, it would be helpful for the readers if the authors briefly outline other open access (OA) initiatives and the OA policies adopted by European and national funding agencies. This addition would strengthen the foundation for the study.
2. In the second paragraph, line 4, the authors wrote, “After two decades of these movements.” It is suggested that the authors mention the names of these movements in brackets to make the sentence clearer for the readers.
3. **Literature review: **The authors have reviewed the literature published up to 2020. However, many studies addressing different aspects of the topic have been published since 2020. The authors are encouraged to incorporate these recent studies to ensure that the literature review reflects the current state of research.
4. **Methodology section: **The author must put the justification to had two separate surveys on different dates.
5. **Results: **The data interpretation is outstanding. However, the authors may also discuss the factors responsible for the findings wherever they have not been addressed. This would provide a more comprehensive interpretation of the results.

If applicable, is the statistical analysis and its interpretation appropriate?

Yes

Is the study design appropriate and is the work technically sound?

Yes

Is the work clearly and accurately presented and does it engage with the current literature?

Yes

Are the conclusions drawn adequately supported by the results?

Yes

Are sufficient details of methods and analysis provided to allow replication by others?

Yes

Are all the source data and materials underlying the results available?

Yes

Reviewer Expertise:

NA

I confirm that I have read this submission and believe that I have an appropriate level of expertise to confirm that it is of an acceptable scientific standard.
